# Resistance‐trained young men exhibit enhanced carotid vasoreactivity during the cold pressor test

**DOI:** 10.1113/EP093966

**Published:** 2026-10-13

**Authors:** Ian Hornby‐Foster, Cory T. Richards, Aimee L. Drane, Freya M. Lodge, Michael Stembridge, Rachel Lord, Zaheer Yousef, Keeron Stone, Barry J. McDonnell, Christopher J. A. Pugh

**Affiliations:** ^1^ Centre for Cardiovascular Research, Innovation and Development Cardiff Metropolitan University Cardiff UK; ^2^ Weston General Hospital Bristol NHS Foundation Trust Weston‐Super‐Mare UK; ^3^ School of Education, Health and Science University of Gloucestershire Gloucester UK; ^4^ Health and Wellbeing Academy, Faculty of Medicine, Health and Life Sciences Swansea University Swansea UK; ^5^ Department of Cardiology University Hospital of Wales Cardiff UK; ^6^ National Cardiovascular Research Network Wales UK; ^7^ School of Life Course and Population Health Sciences King's College London London UK

**Keywords:** cold pressor test, common carotid artery, peak carotid artery reactivity, resistance training

## Abstract

The function of the common carotid artery (CCA) is integral to the regulation of cerebral perfusion. Although endurance training is associated with consistent vascular benefits, the effects of resistance training on CCA function are unclear. The cold pressor test (CPT) assesses CCA vasoreactivity via sympathetic stimulation, with peak CCA vasoreactivity (CAR%_peak_) reflecting integrated endothelial–autonomic vascular function. In this study, we compared CAR%_peak_ during the CPT in resistance‐trained and untrained young men and examined associations with CCA stiffness and haemodynamics. CCA ultrasound was performed in 10 resistance‐trained [24 years (95% confidence interval: 22,26)] and 10 untrained [24 years (22,25)] men at baseline, during a 3‐min CPT and after recovery, with simultaneous haemodynamic assessment via finger plethysmography. CAR%_peak_ was defined as the maximal percentage increase in mean diameter from baseline. Secondary outcomes included CCA stiffness indices and haemodynamics. Both groups exhibited CCA vasodilatation during CPT; however, CAR%_peak_ was greater in resistance‐trained men (*P *= 0.046). The resistance‐trained group also displayed greater increases in systolic diameter at end‐CPT (*P *= 0.033), alongside attenuated pulse pressure (*P *= 0.050; η^2^_p_ = 0.201) and β_1_‐stiffness (*P *= 0.041) responses during CPT. CAR%_peak_ was negatively correlated with baseline β_2_‐stiffness in resistance‐trained men (*r *= −0.655; *P *= 0.040), whereas in untrained control subjects it was positively correlated with peak pulse pressure (*r *= 0.677; *P *= 0.031). In conclusion, resistance‐trained young men exhibit enhanced CCA vasoreactivity and attenuated pulse pressure and stiffness responses to sympathetic stimulation, reflecting favourable adaptations in endothelial–autonomic vascular regulation. These findings highlight the potential of resistance training to improve central vascular function in early adulthood.

## Abstract (highlights)

- **What is the central question of the study?**
  The impact of chronic resistance training on common carotid artery (CCA) vasoreactivity, a key marker of endothelial function, remains poorly defined in young adults. In this study, we investigated whether resistance‐trained men exhibit distinct CCA responses during a sympathetic challenge (cold pressor test) compared with untrained age‐matched control subjects.
- **What is the main finding and its importance?**
  Resistance‐trained men demonstrate augmented CCA vasoreactivity during the cold pressor test compared with age‐matched untrained control subjects. These findings indicate that chronic resistance training promotes superior large‐artery endothelial function, which is likely to optimise the regulation of extracranial and cerebrovascular perfusion during acute sympathetic stress.

## 1 INTRODUCTION

The common carotid artery (CCA) is a major conduit responsible for delivering cerebral blood flow and plays a vital role in regulating cerebral perfusion through its dynamic structural and functional characteristics (Tarumi & Zhang, 2018). Physical activity is a primary modifiable risk factor for optimising vascular health, whereas a sedentary lifestyle promotes a shift in the endothelial phenotype of the CCA towards a more vasoconstrictive, proliferative and pro‐inflammatory state (Seals et al., 2011). Regular endurance exercise promotes favourable CCA adaptations that support cerebral perfusion at rest and during physiological stress, helping to preserve cerebral blood flow and reduce the risk of extracranial and cerebrovascular disease (Seal et al., 2019). These adaptations are evident in both young and older endurance‐trained individuals and include increased CCA diameter, enhanced endothelial function and improved wall compliance, collectively augmenting the capacity of the vessel to buffer pulsatile flow (Seals et al., 2019).

Whilst endurance exercise is consistently associated with favourable central arterial adaptations, whereas the effects of resistance training on central arterial function remain less clear. Although some studies report beneficial reductions in central arterial stiffness (Au et al., 2017; Hornby‐Foster et al., 2025), others report increased stiffness (Kawano et al., 2008; Miyachi, 2013). In contrast, resistance training appears to improve peripheral artery endothelial function more consistently, as evidenced by enhanced flow‐mediated dilatation (FMD) across young (Beck et al., 2013), middle‐aged (Boeno et al., 2020) and adolescent (Yu et al., 2016) populations. However, adaptations in peripheral muscular arteries might not necessarily translate to central elastic arteries, which possess distinct structural and functional characteristics important for buffering pulsatile flow (Leloup et al., 2015). Indeed, although peripheral and central arterial endothelial function are positively correlated (Vallbracht‐Israng et al., 2007), peripheral arteries appear to adapt more rapidly to exercise training than central vessels (Sun et al., 2024). Therefore, direct assessment of the CCA might provide a more specific local measure of central arterial function and adaptation to resistance training.

The cold pressor test (CPT) is an established tool for assessing CCA endothelial function by eliciting sympathetic nervous system activation, alongside transient increases in blood pressure and vascular resistance (van Mil et al., 2017). The resulting CCA diameter response provides insight into local vascular tone, endothelial bioavailability and vasoreactivity to sympathoexcitatory stimuli (van Mil et al., 2017), with responses varying according to arterial health and adrenergic sensitivity. During the CPT, individuals with coronary artery disease exhibit CCA vasoconstriction (Rubenfire et al., 2000), whereas those with cardiovascular disease (CVD) risk factors typically show no change in diameter (Rubenfire et al., 2000; van Mil et al., 2017). Healthy older adults also show limited vasodilatory capacity, possibly reflecting altered α/β‐adrenergic balance (Flück et al., 2017), and young healthy adults elicit significant CCA vasodilatation consistent with preserved endothelial function (Stone et al., 2019). Evidence on resistance training and CCA vasoreactivity is limited, with cross‐sectional (Kawano et al., 2008) and longitudinal (Cortez‐Cooper et al., 2008) studies in middle‐aged adults reporting no effect, suggesting that age‐related CCA remodelling might constrain adaptive responses to sympathetic stimulation. Whether resistance training influences CCA vasoreactivity in young healthy adults, in whom age‐related vascular remodelling is less likely to constrain adaptive responses, remains unknown.

Therefore, the aim of this study was to examine CCA vasoreactivity to sympathetic stimulation using the CPT in young resistance‐trained men and an age‐matched untrained control group. We hypothesised that young resistance‐trained males would demonstrate significant CCA vasodilatation in response to the CPT, similar to untrained control subjects.

## 2 MATERIALS AND METHODS

### 2.1 Ethical approval

Individuals provided written informed consent. Ethical approval was granted by the School of Sport and Health Sciences Research Ethics Committee at Cardiff Metropolitan University (17/3/01S). All procedures were conducted in accordance with the *Declaration of Helsinki* (2008), excluding registration in a public trials database.

### 2.2 Participants

Data collection for this study was conducted as part of a broader programme of investigations examining training‐specific cardiac and carotid artery adaptations in young men in response to a variety of physiological stimuli (Dawkins et al., 2020, 2022; Hornby‐Foster et al., 2025). However, the present study addresses a distinct a priori hypothesis focused explicitly on CPT‐derived CCA vasoreactivity in resistance‐trained and untrained young men. Although previous work from this programme reported data from 14 resistance‐trained participants, only 10 had complete CPT‐derived CCA image datasets suitable for analysis across all required time points. The remaining four were not included because CPT testing was either not performed or incomplete or because the acquired image sequences were not analysable across all time points. Accordingly, 20 young males were included in the present analysis, comprising 10 resistance‐trained [24 years, 95% confidence interval (CI) (22, 26)] and 10 untrained [24 years, 95% CI (22, 25)] individuals.

Habitual physical activity and training status were assessed during the initial laboratory visit using a structured lifestyle and training questionnaire administered by the investigative team. Participants in the resistance‐trained group had engaged exclusively in structured full‐body resistance training for a minimum of 2 years consecutively, with a training frequency of at least three sessions per week and no participation in structured aerobic or endurance exercise. Untrained men had not engaged in regular structured exercise during the preceding 2 years and self‐reported ≤150 min week^−1^ of structured physical activity (Bull et al., 2020). Exclusion criteria included the use of cardioactive drugs and prescribed medications, history of cardiovascular, musculoskeletal or metabolic disease, history of asthma or smoking or any contraindications to exercise. No participants reported using performance‐enhancing drugs.

### 2.3 Study overview

The study implemented a cross‐sectional design and was conducted in a quiet, temperature‐controlled room. Participants attended the laboratory on two occasions. Prior to each visit, they were instructed to abstain from alcohol, caffeine and vigorous exercise for 24 h and to fast for ≥6 h. During the first session, participants completed a one‐repetition maximum (1RM) seated leg press, followed by an incremental cycling test to determine peak oxygen consumption (${\dot{V}}_{O_{2}\mathrm{peak}}$), as previously described (Hornby‐Foster et al., 2025). In brief, the first attempt at 1RM was performed at ∼50% of the participant's weight‐predicted 1RM. The load was then progressively increased over three to five attempts until the participant could complete only a single repetition, thereby reflecting their true 1RM. The ${\dot{V}}_{O_{2}\mathrm{peak}}$ was determined using an incremental cycling test, with initial loads of 50 W for both groups, which were subsequently increased by 20 W every minute until volitional exhaustion. Measurements of ventilatory gas exchange were obtained using a breath‐by‐breath analyser (Oxycon Pro, Jaeger, Warwick, UK). In the absence of a distinct ${\dot{V}}_{O_{2}}$ plateau, ${\dot{V}}_{O_{2}\mathrm{peak}}$ was defined as the highest ${\dot{V}}_{O_{2}}$ achieved over a 30 s consecutive period prior to volitional exhaustion. At the second visit, short‐axis ultrasound images of the CCA were obtained at baseline, during a CPT and following a recovery period.

### 2.4 Experimental procedures

#### 2.4.1 CPT

Participants completed a 3‐min CPT by placing their left hand into ice water (4°C) whilst supine. This is a widely accepted and validated model to stimulate the sympathetic nervous system, and this particular protocol has previously been shown to elicit transient increases in sympathetic activation and evoke haemodynamic and arterial responses (Thijssen et al., 2006; van Mil et al., 2017). Furthermore, 3‐min CPT protocols ensure that CCA responses are correlated with the period of maximal sympathetic stimulation, normally present during 90 s into the CPT (van Mil et al., 2017). A hand‐immersion protocol was selected because it represents the most widely validated and reproducible model for eliciting an acute, systemic sympathetic pressor response (van Mil et al., 2017). CCA ultrasound measurements were obtained prior to the onset of the CPT (baseline), 90 s following hand immersion (mid), immediately prior to removing the hand (end; 180 s) and 60 s after hand removal (recovery; 240 s). Participants were instructed not to speak and to breathe normally during the CPT and were verbally encouraged throughout. The CPT was performed without prior familiarisation to the ice water stimulus, to preserve the acute cold‐induced sympathetic response and avoid habituation that could influence baseline or CPT‐evoked measurements. Beat‐by‐beat blood pressure, stroke volume (SV), cardiac output ($\dot{Q}$) and total peripheral resistance (TPR) were recorded continuously via finger plethysmography (FinometerPro, FMS, Groningen, The Netherlands) on the contralateral hand. These blood pressure data were then calibrated against manual baseline brachial blood pressure measurements taken prior to CPT. All stroke volume data were adjusted to body surface area [stroke volume index (SVI)], which was calculated using the Du Bois formula (Cattermole et al., 2017).

#### 2.4.2 CCA ultrasound imaging

Short‐axis grey‐scale cine loops of the right CCA were acquired on a commercially available high‐resolution ultrasound system (Vivid q, GE Healthcare, Chalfont, UK), using a 12 MHz multi‐frequency linear array probe. Images were recorded ∼2 cm distal to the carotid bifurcation for six consecutive cardiac cycles. Cardiac cycles were measured continuously using a three‐lead ECG connected to the ultrasound machine. Frame rate (92.3 frames s^−1^), imaging depth and probe orientation were standardised for all participants, and all images were acquired by a single trained technician.

#### 2.4.3 Common carotid diameter and vasoreactivity

CCA diameters were obtained using M‐mode ultrasound. Systolic and diastolic diameters were defined as the maximal and minimal arterial diameters during and following pulsatile ejection, respectively. These were averaged over six cardiac cycles using callipers placed on the inner‐to‐inner anteroposterior CCA walls. Mean diameter, calculated as the average of systolic and diastolic diameters for each cycle, was recorded at baseline, during both CPT time points and after recovery. Mean diameter was used as a composite measure of vessel calibre reflecting the reactive change in diameter throughout the cardiac cycle in response to sympathetic stimulation. This provides an index of CCA vasoreactivity (van Mil et al., 2017), which is an established marker of local CCA endothelial function (Stone et al., 2019). Peak CCA vasoreactivity (CAR%_peak_) was defined as the greatest percentage change in mean diameter from baseline, observed at either the mid‐point or end of the CPT protocol for each participant.

#### 2.4.4 CCA stiffness parameters

To characterise local CCA stiffness, the distensibility coefficient (DC) was calculated using the formula: DC = (2DΔD + (ΔD)^2^)/(D^2^ΔP) (van Bortel et al., 2002); where Δ*D* represents distension, *D* is the diastolic diameter, and Δ*P* is pulse pressure (PP). This reflects the relative change in CCA diameter during the cardiac cycle for a stroke change in blood pressure and has demonstrated a lower intersession coefficient of variation in comparison to alternative stiffness indices at the CCA (van Bortel et al., 2002). We also used the β_1_‐stiffness index [β_1_‐stiffness; in arbitrary units (a.u.)] and β_2_‐stiffness index (β_2_‐stiffness; in arbitrary units) to characterise CCA stiffness, which were calculated using established formulas from our previous investigations (Hornby‐Foster et al., 2025; Pugh et al., 2018). β_2_‐Stiffness was derived from peak circumferential strain (PCS), assessed via two‐dimensional speckle‐tracking software (EchoPac v.112, GE Vingmed Ultrasound, Horten, Norway) to quantify global arterial wall deformation across the cardiac cycle, as described in our earlier studies (Hornby‐Foster et al., 2025; Pugh et al., 2018; Talbot et al., 2020).

### 2.5 Statistical analysis

An a priori sample‐size estimation for CAR%_peak_ was informed by effect size and variance estimates from two previous CPT studies: one examining the effect of sex differences in CAR%_peak_ in young adults (Stone et al., 2019) and one investigating age‐related differences in CAR%_peak_ (Flück et al., 2017). These studies reported mean absolute between‐group differences in CAR%_peak_ of 5.0% between healthy young and older adults (Cohen's *d* effect size = 1.66) and ∼2.6% between healthy young men and women (*d* = 1.18). Based on the pooled variance estimate most relevant to the young adult demographic, an independent two‐group comparison with 20 participants in total, comprising 10 participants per group, was estimated to provide 80% power to detect a minimum between‐group difference of ∼3.0% in CAR%_peak_ (*d* = 1.32) using a two‐sided α‐level of 0.05. All data were checked and confirmed for normality of distribution using a Shapiro–Wilk test. Differences in baseline descriptive characteristics between groups were assessed using independent *t*‐tests. To evaluate responses across the CPT, a two‐way repeated‐measures ANOVA was conducted to determine the main effects of time, group and any interaction between these factors (group × time). ANOVAs were conducted using relative change data, expressed as a percentage change from baseline at each protocol time point. When a significant interaction was identified, *post hoc* pairwise comparisons were performed with Bonferroni adjustments. An additional independent *t*‐test was performed to assess the differences in CAR%_peak_ between groups. Pearson's correlation was used to assess the exploratory relationship between CAR%_peak_ and baseline haemodynamics and CCA stiffness indices, in addition to the peak percentage changes in these variables during the CPT. Effect sizes were calculated to contextualise the magnitude of the findings close to significance, using partial eta squared (η^2^_p_) for ANOVA and Cohen's *d* for pairwise comparisons. Analyses were carried out using SPSS (v.27 for Windows, SPSS Inc., Chicago, IL, USA), and results are reported as mean percentage changes from baseline values [95% confidence intervals]. Statistical significance was set a priori at *P* < 0.05.

## 3 RESULTS

### 3.1 Descriptive characteristics

Baseline descriptive characteristics, haemodynamics, CCA stiffness indices and diameters are presented in Table 1. In line with our study design, the resistance‐trained group demonstrated greater 1RM and training frequency compared with control subjects (*P* < 0.001). They also had a higher body mass, body mass index (*P* < 0.001) and body surface area (*P* = 0.003). Baseline heart rate was lower in the resistance‐trained group (*P* = 0.001), and SVI was higher (*P* = 0.002). Finally, baseline systolic blood pressure (SBP) was significantly higher in resistance‐trained participants (*P* = 0.021). No other blood pressure variables differed between the groups, nor were there differences in ${\dot{V}}_{O_{2}\mathrm{peak}}$, fat‐free mass or body fat percentage (*P* > 0.196). Likewise, CCA diameters (*P* > 0.065) and stiffness indices (*P* > 0.120) did not differ between groups.

**TABLE 1:** Baseline descriptive characteristics, haemodynamics, common carotid artery stiffness indices and diameters in resistance‐trained and untrained men.

Measured variables	Resistance‐trained (*n *= 10)	Untrained (*n *= 10)	*P*‐value
**Characteristics**			
Age, years	24 [22, 26]	24 [22, 25]	0.471
Height, cm	182.4 [178, 186.4]	181 [174, 187.9]	0.703
Body mass, kg	89.7 [84, 95.7]	73.5 [68.3, 78.7]	**<0.001**
BMI, kg.m^−2^	27.5 [25.7, 28.3]	22.6 [20.5, 24.7]	**<0.001**
Body fat, %	13.5 [11, 16.1]	14.7 [9.7, 19.8]	0.208
FFM, kg	69.2 [53.2, 85.2]	62.2 [58, 66.4]	0.482
Body surface area, m^2^	2.12 [2.03, 2.21]	1.93 [1.84, 2.02]	**0.003**
${\dot{V}}_{O_{2}\mathrm{peak}}$, mL kg^−1^ min^−1^	39.3 [35.9, 42.7]	41 [38, 44.2]	0.196
1RM, kg	448 [424, 471]	217 [177, 256]	**<0.001**
Training history, years	6.2 [4.8, 7.5]		
Training frequency, session week^−1^	5.1 [4.6, 5.5]	0.7 [0.3, 1.0]	**<0.001**
**Haemodynamics**			
SBP, mmHg	127 [123, 131]	121 [116, 126]	**0.021**
DBP, mmHg	78 [70, 85]	75 [71, 79]	0.422
PP, mmHg	49 [42, 56]	46 [40, 51]	0.156
MAP, mmHg	94 [88, 100]	91 [87, 94]	0.368
SV, mL	141 [129, 153]	112 [106, 117]	**<0.001**
SVI, mL m^−2^	67 [51, 72]	57 [55, 60]	**0.002**
HR, beats min^−1^	56 [50, 62]	68 [63, 72]	**0.001**
$\dot{Q}$, L min^−1^	7.9 [6.9, 8.8]	7.6 [7.1, 8]	0.252
TPR, mmHg min mL^−1^	12.3 [10.6, 13.9]	12 [11.2, 12.8]	0.362
**CCA diameters**			
DD, mm	5.72 [5.42, 6]	5.78 [5.65, 5.9]	0.341
SD, mm	6.45 [6.14, 6.76]	6.59 [6.41, 6.76]	0.193
MD, mm	6.1 [5.78, 6.39]	6.18 [6.03, 6.33]	0.255
**CCA stiffness indices**			
PCS, %	12.1 [10.9, 13.2]	11.5 [10.4, 12.7]	0.249
DC, ×10^−^^3^ mmHg^−1^	5.88 [4.6, 7.1]	6.8 [5.6, 7.9]	0.121
β_1_, a.u.	3.97 [2.98, 4.97]	3.43 [2.89, 3.97]	0.146
β_2_, a.u.	2.62 [2.5, 2.74]	2.64 [2.53, 2.76]	0.393

*Note*: Data are presented as absolute means [95% confidence interval]. Abbreviations: 1RM, one‐repetition maximum; BMI, body mass index; DBP, diastolic blood pressure; DC, distensibility coefficient; DD, diastolic diameter; FFM, fat free mass; HR, heart rate; MAP, mean arterial pressure; MD, mean diameter; PCS, peak circumferential strain. PP, pulse pressure; $\dot{Q}$, cardiac output; SBP, systolic blood pressure; SD, systolic diameter; SVI, stroke volume index; TPR, total peripheral resistance; ${\dot{V}}_{O_{2}\mathrm{peak}}$, peak oxygen consumption; β_1_, β_1_‐stiffness index; β_2_, β_2_‐stiffness index.

### 3.2 CCA vasoreactivity during CPT

A significant effect of time was observed for both systolic (*P* < 0.001; Figure 1b) and mean (*P = *0.002; Figure 1c) diameter. In both groups, values increased at the midpoint and end point of the CPT and returned to baseline during recovery. However, no effect of time was observed for diastolic diameter during the CPT (*P* = 0.071; Figure 1a). No group effects were observed for mean (*P* = 0.331), systolic (*P =* 0.106) or diastolic diameters (*P =* 0.808). A close to significant group × time interaction was observed for systolic diameter (*P* = 0.051; effect size η^2^_p_ = 0.174), with *post hoc* pairwise comparisons revealing a greater increase at the end point in the resistance‐trained group compared with untrained control subjects (*P* = 0.033). However, there were no interaction effects for mean (*P* = 0.242) or diastolic (*P =* 0.672) diameters. Finally, the resistance‐trained group exhibited a significantly greater CAR%_peak_ than untrained control subjects (7.7% [95% CI: 3.5, 11.9] vs. 3.8% [95% CI: 1.7, 5.9]; *P* = 0.046; Cohen's *d* effect size = 0.84; Figure 2).

**FIGURE 1:**
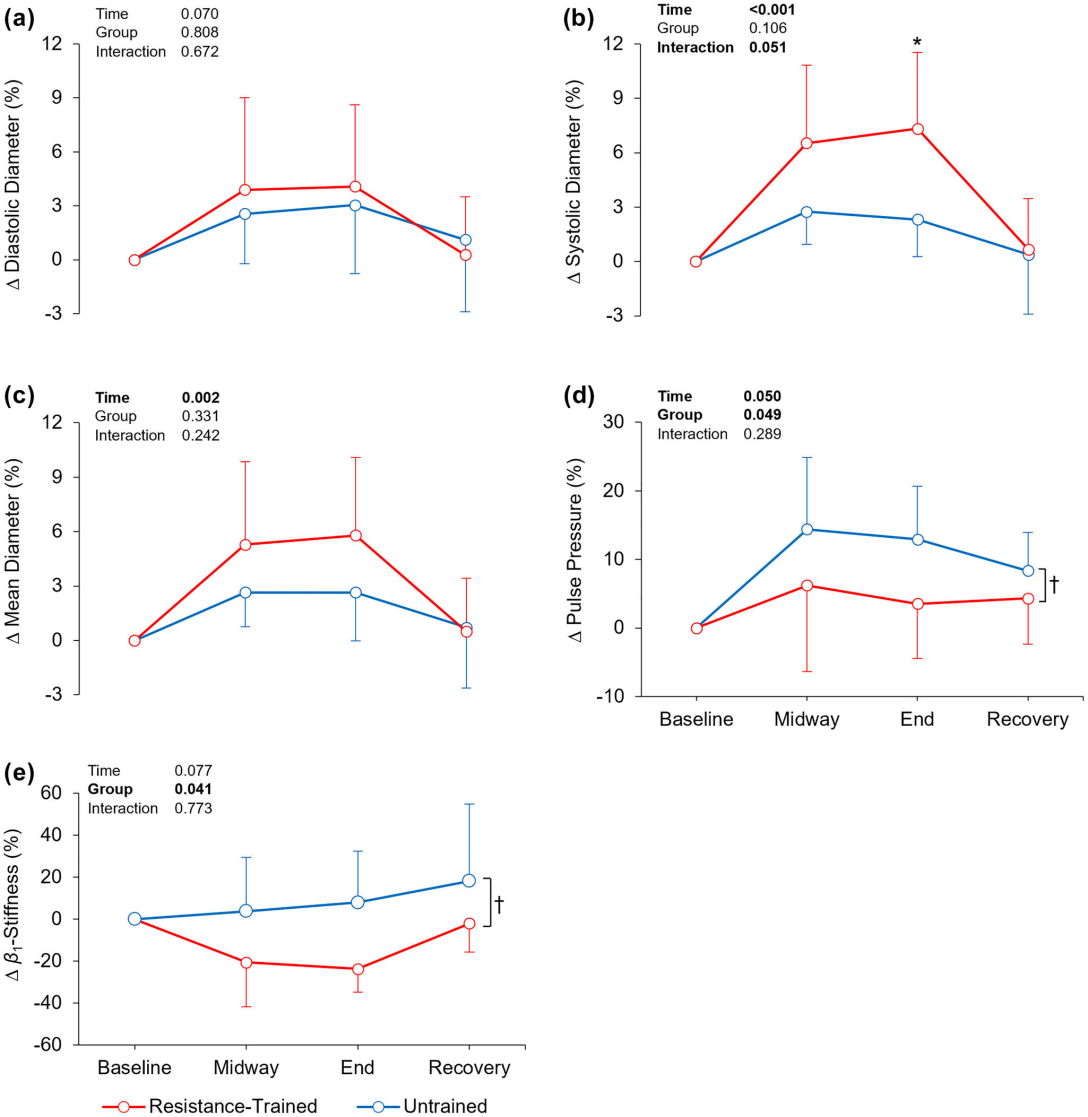
Diastolic diameter (a), systolic diameter (b), mean diameter (c), pulse pressure (d) and β_1_‐stiffness (e) at baseline, during (mid and end) and after a 3 min cold pressor test in resistance‐trained (red, *n *= 10) and untrained (blue, *n *= 10) young men. Data are presented as the mean percentage change from baseline, and error bars represent 95% confidence interval. Individual data points can be found in the Appendix, Table A1. Two‐way ANOVA effects for time and group and group × time interactions are presented. *P < 0.05 pairwise difference between resistance‐trained vs. untrained groups at the same time point. †Significant between‐group difference.

**FIGURE 2:**
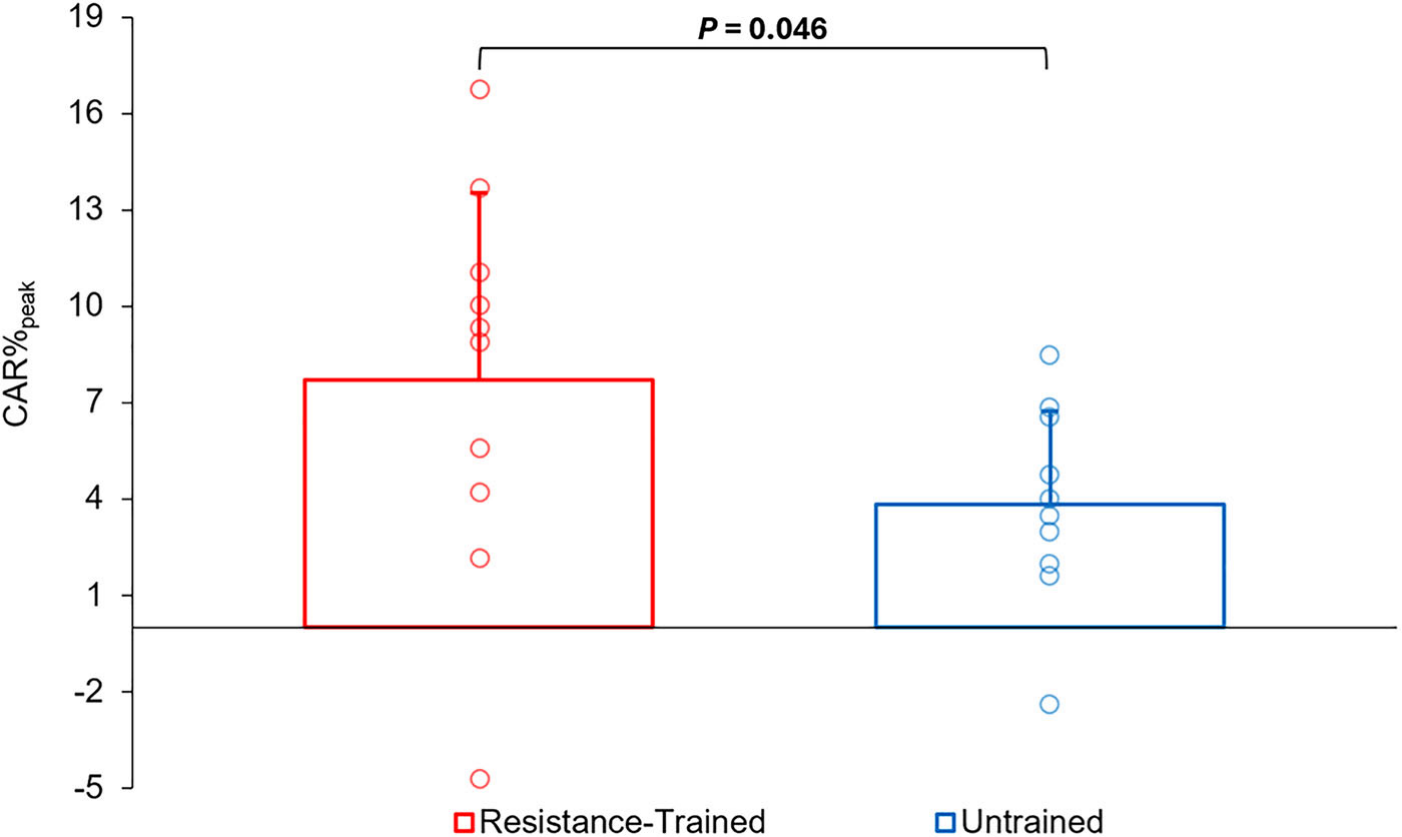
Peak common carotid artery vasoreactivity (CAR%_peak_, presented as the peak percentage change from baseline values) during a 3 min cold pressor test in young resistance‐trained (red, *n *= 10) and untrained (blue, *n *= 10) men. Data are presented as means with individual participant data points, and error bars represent the 95% confidence interval. Independent sample *t*‐test: *P < 0.05 difference between groups for CAR%_peak_.

### 3.3 CCA stiffness and haemodynamic responses during CPT

CCA stiffness and haemodynamics during the CPT are displayed in Table 2. A significant effect of time was observed in DC (*P* = 0.013), PCS (*P* = 0.047; η^2^_p_ = 0.158), all blood pressure variables (*P* < 0.05) and TPR (*P* = 0.006). In both groups, blood pressures, TPR and DC increased, and PCS decreased, in response to the CPT and returned to baseline values in recovery. There were no time effects observed for β_1_‐stiffness, heart rate, SVI or $\dot{Q}$ (*P *> 0.060). A significant group effect was observed for PP (*P* = 0.050; η^2^_p_ = 0.201) and β_1_‐stiffness (*P* = 0.041; Figure 1e), with the resistance‐trained group demonstrating an attenuated rise in PP during the CPT and consistently lower β_1_‐stiffness values throughout the stimulus compared with untrained control subjects. A significant group × time interaction was observed for PCS (*P* = 0.041) and β_2_‐stiffness (*P* = 0.040); however, *post hoc* pairwise comparisons revealed no significant group differences at any time point during the CPT (PCS, *P* > 0.269; β_2_‐stiffness, *P* > 0.148). No further effects of time, group or group × time interactions were detected for any CCA stiffness or haemodynamic variable.

**TABLE 2:** Haemodynamics and common carotid artery stiffness parameters, expressed as percentage change from baseline values, during a 3 min cold pressor test and recovery period in resistance‐trained and untrained young men.

	Resistance trained (*n *= 10)			Untrained (*n *= 10)			*P*‐value		
Measured variable (% change)	Midway	End	Recovery	Midway	End	Recovery	Time effect	Group effect	Group × time interaction
**Haemodynamics**									
SBP, mmHg	10.5 [5, 16]	6.6 [3, 10.2]	5.8 [2.7, 8.9]	15 [9.4, 20.5]	12.8 [8.4, 17.2]	7 [3.3, 10.7]	**<0.001**	0.128	0.081
DBP, mmHg	13.9 [8.4, 19.3]	9 [5.3, 12.6]	7.2 [3, 11.3]	15.8 [10, 21.5]	13.2 [7.7, 18.7]	6.4 [1.5, 11.3]	**<0.001**	0.526	0.103
PP, mmHg	6.2 [−1.2, 13.7]	3.5 [−2, 9.1]	4.3 [0.3, 8.3]	14.4 [5.4, 23.4]	12.9 [7.2, 18.6]	8.4 [3.6, 13.1]	**0.050**	**0.049**	0.218
MAP, mmHg	12.3 [7, 17.5]	7.8 [4.5, 11.2]	6.5 [3, 9.9]	15.4 [10, 20.8]	13 [8.1, 17.9]	6.7 [2.4, 10.9]	**<0.001**	0.282	0.068
SVI, mL m^−2^	−0.6 [−6.5, 5.3]	2.8 [−3.8, 9.4]	0.9 [−3.4, 5.1]	3.8 [−3.9, 11.6]	6 [−2.4, 14.3]	7.5 [1.7, 13.2]	0.064	0.348	0.208
HR, beats min^−1^	0 [−10, 10]	−0.6 [−10.7, 9.6]	0.8 [−5.9, 7.5]	8.2 [−0.3, 16.6]	7.9 [−0.5, 16.2]	3.2 [−3.7, 10]	0.588	0.197	0.288
$\dot{Q}$, L min^−1^	−1.3 [−9.1, 6.6]	2.2 [−10, 14.8]	1.4 [4.4, 7.3]	11.6 [2.6, 20.7]	13.1 [5, 21.3]	12 [4.7, 19.3]	0.561	0.563	0.634
TPR, mmHg min mL^−1^	15.3 [2.8, 27.8]	8.2 [5.1, 21.6]	5.7 [−2, 13.7]	3.9 [−4, 11.7]	0.5 [−7.8, 8.8]	−4.9 [−11.7, 1.9]	**0.006**	0.817	0.727
**CCA stiffness parameters**									
PCS, %	−14 [−22.5, −5.5]	−13.7 [−23.4, −4]	−1.8 [−13, 9.7]	−13.6 [−27 −0.6]	−10 [−23.9, 4]	−12 [−27.3, 3.3]	**0.047**	0.788	**0.041**
β_2_, a.u.	4.9 [1.5, 8.2]	5.2 [0.9, 9.5]	0.8 [−4.3, 5.8]	6.2 [0.6, 11.8]	4.9 [1.3, 11.2]	6.4 [−0.4, 13.2]	0.223	0.473	**0.041**
DC, ×10^−^^3^ mmHg^−1^	28.5 [−1.3, 58]	21.9 [7.2, 36.6]	−5.8 [−18, 6.3]	−6.9 [−30.8, 17]	−8 [−33.8, 17.8]	−10.7 [−30.5, 9]	**0.013**	0.063	0.062

*Note*: Data are presented as percentage changes from baseline values [95% confidence interval]. Abbreviations: DBP, diastolic blood pressure; DC, distensibility coefficient; HR, heart rate; MAP, mean arterial pressure; PCS, peak circumferential strain; PP, pulse pressure; $\dot{Q}$, cardiac output; SBP, systolic blood pressure; SVI, stroke volume index; TPR, total peripheral resistance; β_2_, β_2_‐stiffness index.

### 3.4 Correlates of CAR%_peak_

No significant correlations were observed when data were pooled across both groups. When conducting subgroup correlation analysis, in the resistance‐trained group only, there was a significant negative correlation between CAR%_peak_ and baseline β_2_‐stiffness [Figure 3a,b; *r* = ‐0.655; *P* = 0.041 (vs. untrained group: *r* = 0.414; *P* = 0.234)] and the peak percentage increase in TPR [*r* = ‐0.731; *P* = 0.016 (vs. untrained group: *r* = 0.088; *P* = 0.812)], whereas, in untrained control subjects only, there was a significant positive correlation between CAR%_peak_ and the peak percentage increase in systolic [*r* = 0.722; *P* = 0.018 (vs. resistance‐trained group: *r* = ‐0.397; *P* = 0.256]) and PPs [Figure 3c,d; *r* = 0.677; *P* = 0.031 (vs. resistance‐trained group: *r* = ‐0.216; *P* = 0.549)]. No other significant correlations were observed between CAR%peak and the remaining pressure variables or CCA stiffness indices at baseline or during peak response to the CPT.

**FIGURE 3:**
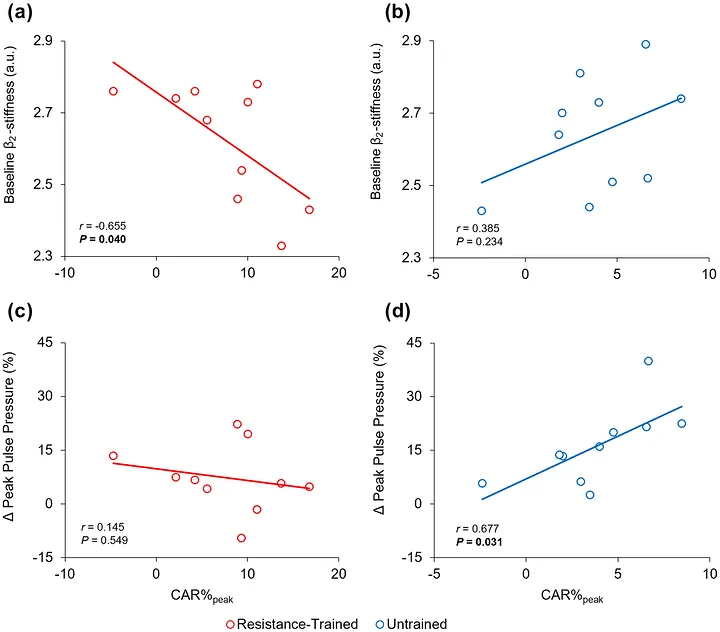
Relationship between peak common carotid artery vasoreactivity (CAR%_peak_) and baseline β_2_‐stiffness [(a) resistance trained (red, *n *= 10); and (b) untrained (blue, *n *= 10)], and the change in peak pulse pressure during a 3‐min cold pressor test (c, d).

## 4 DISCUSSION

The aim of this study was to examine CCA vasoreactivity to sympathetic stimulation, assessed via the CPT, in young resistance‐trained men compared with age‐matched untrained control subjects. To our knowledge, this is the first study to investigate CCA vasoreactivity to a CPT in this population. In line with our primary hypothesis, untrained men exhibited a normal vasodilatory response; however, resistance‐trained men demonstrated a significantly greater CAR%_peak_ during the CPT. These findings indicate that resistance‐training enhances CCA vasoreactivity, reflecting favourable central arterial adaptations and improved endothelial–autonomic regulation.

Our finding of enhanced CAR%_peak_ in young resistance‐trained men contrasts with previous reports of no effect of resistance training on CCA vasoreactivity in middle‐aged cohorts (Cortez‐Cooper et al., 2008; Kawano et al., 2008), suggesting that age might modulate central artery adaptability to resistance training. Consistent with this, CAR%_peak_ in the present cohort (7.7%) exceeded values reported in middle‐aged resistance‐trained groups (∼4%) (Cortez‐Cooper et al., 2008; Kawano et al., 2008), young untrained adults (∼4%) (Stone et al., 2019; van Mil et al., 2017) and older or CVD populations, in which blunted or constricted responses are common (Flück et al., 2017; Rubenfire et al., 2000; van Mil et al., 2017). This heightened vasoreactivity is likely to reflect greater endothelial nitric oxide bioavailability (Green et al., 2011), shear stress responsiveness (Thijssen et al., 2011), improved local buffering (van Mil et al., 2017) and favourable adrenergic signalling adaptations (Rubenfire et al., 2000) in our cohort. In contrast, age‐related stiffening, elevated basal vascular tone and endothelial impairment (Flück et al., 2017) are likely to explain the blunted responses in middle‐aged cohorts (Kawano et al., 2008; Cortez‐Cooper et al., 2008), whereas young arteries remain fully responsive, enabling unconstrained optimal adaptation to resistance training.

Evidence from studies directly examining CAR%_peak_ in young resistance‐trained populations remains limited. However, peripheral vascular studies indicate that resistance training can enhance endothelial function in adolescents and young adults (Beck et al., 2013; Boeno et al., 2020; Yu et al., 2016), suggesting that the present findings might reflect broader endothelial adaptation across peripheral and central vascular beds. Although extrapolation from peripheral FMD to the CCA requires caution, endothelial responses across vascular beds are known to be related. For example, brachial FMD is strongly correlated with coronary endothelium‐dependent vasodilatation in response to acetylcholine (Broxterman et al., 2019), and CAR%_peak_ has been associated with coronary artery responses during CPT (van Mil et al., 2017). Given that the CAR response to CPT is at least partly endothelium mediated (Tousoulis et al., 1997; van Mil et al., 2017), the elevated CAR%_peak_ observed here might reflect enhanced systemic endothelial function in resistance‐trained men.

Although CAR%_peak_ was augmented in the resistance‐trained group, mean diameter change did not differ between groups at either fixed time point, consistent with observations in young, healthy adults (Stone et al., 2019). CAR%_peak_ captures the maximal dynamic vasoreactivity of each participant, irrespective of when peak dilatation occurs, whereas mean diameter change measured at a fixed time point might mask group differences attributable to interindividual variability in the timing of peak dilatation during the CPT. Notably, only 30% of resistance‐trained and 50% of untrained participants peaked at 90 s, challenging the conventional assumption that maximal dilation occurs at 90 s of CPT (van Mil et al., 2017). Similar interindividual variability has been reported previously (Flück et al., 2017; Tymko et al., 2017), probably reflecting differences in endothelial–autonomic responsiveness. Unlike mean and diastolic diameter, systolic diameter was significantly elevated in the resistance‐trained group at the end of the CPT, suggesting enhanced systolic vasodilatory capacity, which might reflect adaptation to the repeated elevated systolic pressures experienced during resistance training.

Basal CCA stiffness appears to influence the CAR%_peak_ response in the resistance‐trained group. Baseline β_2_‐stiffness was negatively correlated with CAR%_peak_, indicating that greater arterial compliance is associated with a larger CPT‐induced vasodilatory response. The resistance‐trained group also exhibited an attenuated rise in β_1_‐stiffness during CPT, suggesting preserved buffering of transient pressure loading without excessive local arterial stiffening. This contrasts with findings in middle‐aged resistance‐trained men, in whom β_1_‐stiffness increases more markedly during CPT than in untrained control subjects (Kawano et al., 2008). Notably, the elevated CAR%_peak_ occurred despite an attenuated increase in PP, suggesting that enhanced vasoreactivity was not driven simply by a larger pressor stimulus. Together, these responses indicate a more compliant vascular phenotype in the resistance‐trained cohort, facilitating greater vasodilatory responsiveness during sympathetic stimulation. Although endurance training promotes functional sympatholysis through blunted α_1_/α_2_‐mediated vasoconstriction and enhanced β‐mediated vasodilatation (Mortensen et al., 2014), the effects of resistance training on adrenergic regulation remain less clear. The greater CAR%_peak_ and blunted β_1_‐stiffness response observed here might reflect adaptations in local adrenergic responsiveness and/or endothelial nitric oxide bioavailability arising from repeated episodic exposure to elevated pressure, pulsatile flow and shear stress during resistance exercise (Green et al., 2017).

Despite higher resting SBP in the resistance‐trained group, the SBP response to CPT was similar between groups, while the PP increase was attenuated. This indicates that elevated resting SBP did not impair CCA buffering of acute pressure loading during sympathetic stress. The enhanced CAR%_peak_ observed alongside an attenuated PP response suggests greater local vasoreactivity, consistent with endothelium‐mediated dilatation rather than passive pressure‐mediated distension. Correlation analysis further supports this interpretation, because CAR%_peak_ was positively associated with PP in untrained men but not in resistance‐trained men, indicating a degree of functional decoupling from haemodynamic loading. This aligns with our previous observations of augmented CCA two‐dimensional circumferential strain at rest and in response to isometric hand‐grip exercise in resistance‐trained men (Hornby‐Foster et al., 2025), consistent with enhanced arterial compliance and dynamic responsiveness to acute physiological stress. Together, these findings suggest that resistance‐trained men maintain rapid vasodilatory and buffering responses to acute sympathetic pressor stress, which might have favourable implications for carotid–cerebral haemodynamics. Indeed, blunted PP responses and enhanced CCA function have been associated with lower carotid and cerebral pulsatility, reduced plaque formation and lower stroke risk (Lambiase et al., 2014; Matthews et al., 1998; Mitchell et al., 2011; Yang et al., 2018).

This study has limitations that should be considered. First, our findings are limited to young male participants. Given reported sex differences in CCA vasoreactivity during CPT (Stone et al., 2019), resistance‐trained females might exhibit distinct baseline characteristics and divergent responses. Future studies should examine the interaction between sex and resistance training on CCA vasoreactivity. Second, this study did not use continuous diameter measurements used in some CAR% CPT studies (Flück et al., 2017; Stone et al., 2019; Tymko et al., 2017; van Mil et al., 2017). Instead, cine loops were acquired at preset time points, consistent with previous carotid vasoreactivity studies (Kawano et al., 2008; Londono et al., 2014; Rubenfire et al., 2000), including at 90 s of CPT, a commonly used index of CCA peak dilatation (Thijssen et al., 2011; van Mil et al., 2017). Third, although appropriately powered for the primary CAR%_peak_ analysis, secondary correlations should be interpreted cautiously given the modest cohort size. Fourth, we did not capture detailed training‐intensity history and therefore cannot determine the influence of overall resistance‐training load. Finally, concurrent measurements of CCA blood flow and shear rate were not obtained, which might have provided additional mechanistic insight into the divergent vasodilatory responses between groups. However, previous studies in young adults indicate that CAR%_peak_ during the CPT is not directly related to changes in carotid blood flow or shear rate but is more strongly associated with changes in mean arterial pressure (Stone et al., 2019). In the present study, mean arterial pressure responses were similar between groups, whereas the rise in PP was attenuated in resistance‐trained men. Given the divergent blood pressure associations, the augmented CAR%_peak_ in the resistance‐trained group is likely to reflect intrinsic vascular mechanisms beyond passive local haemodynamics.

## 5 CONCLUSION

In conclusion, this study demonstrates, for the first time, that young resistance‐trained men exhibit significantly greater CAR%_peak_ during CPT compared with untrained control subjects. They also exhibited blunted PP and β_1_‐stiffness rises during CPT, suggesting a functional decoupling from passive pressure‐mediated dilatation. Together, these features are likely to reflect integrated adaptations induced by resistance training, culminating in a protective CCA phenotype. Finally, our findings build on a broader conceptual framework that resistance training enhances not only structural integrity (Hornby‐Foster et al., 2025), but also functional responsiveness of the CCA during acute sympathetic and haemodynamic stress.

## AUTHOR CONTRIBUTIONS

Ian Hornby‐Foster and Christopher J. A. Pugh conceived and designed the research; Ian Hornby‐Foster, Aimee L. Drane, Rachel Lord, Cory T. Richards, Freya M. Lodge and Michael Stembridge performed experiments; Ian Hornby‐Foster analysed data; Ian Hornby‐Foster and Christopher J. A. Pugh interpreted results of experiments; Ian Hornby‐Foster prepared figures; Ian Hornby‐Foster drafted manuscript; Ian Hornby‐Foster, Aimee L. Drane, Rachel Lord, Cory T. Richards, Freya M. Lodge, Zaheer Yousef, Michael Stembridge, Keeron Stone, Barry J. McDonnell and Christopher J. A. Pugh edited and revised manuscript. All authors approved the final version of manuscript and agree to be accountable for all aspects of the work in ensuring that questions related to the accuracy or integrity of any part of the work are appropriately investigated and resolved. All persons designated as authors qualify for authorship, and all those who qualify for authorship are listed.

## CONFLICT OF INTEREST

No conflicts of interest, financial or otherwise, are declared by the authors.

## FUNDING INFORMATION

No funding and/or grant was received for this research.

## GENERATIVE AI STATEMENT

No AI tools were used in the conception, study design, data collection, analysis, interpretation of findings or manuscript writing.

## Data Availability

Data supporting the findings of this study are available from the corresponding author upon reasonable request.

## 1

**TABLE A1:** Individual data points for the group means in Figure 1.

Subject	Figure 1a: %change in DD			Figure 1b: %change in SD			Figure 1c: %change in MD			Figure 1d: %change in PP			Figure 1e: %change in β_1_		
	MID	END	REC	MID	END	REC	MID	END	REC	MID	END	REC	MID	END	REC
**Untrained**															
1	−0.18	−0.37	1.85	5.82	4.83	4.99	2.97	2.36	3.50	4.17	6.25	4.17	−40.03	−45.10	−26.42
2	−3.39	5.08	6.95	2.22	2.07	3.99	−0.39	3.48	5.37	2.56	2.56	−2.56	25.94	1.44	23.72
3	3.68	10.53	7.89	5.26	6.66	8.05	4.52	8.47	7.98	22.45	14.29	8.16	−13.46	19.63	−0.87
4	5.42	−3.67	−6.64	4.15	−1.38	−5.23	4.75	−2.45	−5.89	18.00	20.00	18.00	11.64	−14.99	−6.06
5	1.02	−3.39	−1.69	2.86	1.65	1.35	1.99	−0.72	−0.08	13.33	8.89	13.33	−16.42	−35.18	−22.48
6	2.36	2.69	5.89	1.03	1.03	0.15	1.65	1.81	2.83	13.79	10.34	3.45	3.81	38.86	81.70
7	−1.66	−2.16	−3.32	−3.17	−2.60	0.00	−2.47	−2.39	−1.54	−5.77	5.77	11.54	−2.39	1.35	−12.18
8	2.95	10.40	0.52	2.73	3.19	0.00	2.83	6.55	0.24	19.61	21.57	5.88	−0.38	69.42	−4.46
9	5.49	4.42	6.37	2.72	2.57	−2.57	3.99	3.42	1.55	16.00	12.00	4.00	32.33	19.82	134.11
10	9.90	6.77	−6.77	3.82	5.19	−7.02	6.66	5.93	−6.90	40.00	27.50	17.50	88.54	24.64	15.48
**Resistance** **trained**															
1	11.34	4.09	−1.86	15.75	7.63	1.95	13.69	5.98	0.17	5.71	2.86	0.00	−39.18	−23.05	−24.93
2	4.31	9.69	0.00	10.19	12.26	0.00	7.43	11.05	0.00	−6.15	−1.54	0.00	−44.02	−32.01	−11.15
3	3.16	−3.49	−1.00	7.74	−1.34	−1.64	5.58	−2.36	−1.34	4.26	0.00	0.00	−39.08	−30.82	2.67
4	−3.14	1.57	3.32	2.19	6.56	1.72	−0.33	4.20	2.47	6.67	−1.67	6.67	−45.82	−35.92	20.91
5	−9.52	−8.76	−7.68	−4.82	−0.99	−4.39	−7.07	−4.72	−5.97	13.46	9.62	3.85	−43.80	−53.10	−34.83
6	1.88	7.91	2.64	5.96	10.60	3.48	4.05	9.34	3.08	−11.90	−9.52	−2.38	33.70	−25.84	−9.90
7	15.19	14.20	10.26	14.99	19.05	10.05	15.08	16.76	10.15	4.76	0.00	2.38	18.58	−18.33	21.05
8	5.28	7.84	−2.56	4.01	9.81	−2.23	4.60	8.89	−2.38	22.22	11.11	14.81	10.20	−2.56	−1.15
9	11.82	6.08	2.30	8.46	7.03	0.72	10.03	6.58	1.45	19.51	17.07	12.20	32.32	−5.03	18.18
10	−1.42	1.59	−2.65	0.78	2.64	−2.95	−0.25	2.15	−2.81	3.70	7.41	5.56	−21.23	−10.05	−1.18

*Note*: Data are presented as percentage changes from baseline values for each individual included within the study sample.

Abbreviations: DD, diastolic diameter; MD, mean diameter; PP, pulse pressure; SD, systolic diameter; β_1_, β_1_‐stiffness index.

## References

[eph70452-bib-0001] Ashby, J. W. , & Mack, J. J. (2021). Endothelial control of cerebral blood flow. The American Journal of Pathology, 191(11), 1906–1916. 10.1016/j.ajpath.2021.02.023 33713686

[eph70452-bib-0002] Au, J. S. , Oikawa, S. Y. , Morton, R. W. , MacDonald, M. J. , & Phillips, S. M. (2017). Arterial stiffness is reduced regardless of resistance‐training load in young men. Medicine and Science in Sports and Exercise, 49(2), 342–348. 10.1249/MSS.0000000000001106 27669442

[eph70452-bib-0003] Beck, D. T. , Casey, D. P. , Martin, J. S. , Emerson, B. D. , & Braith, R. W. (2013). Exercise training improves endothelial function in young prehypertensives. Experimental Biology and Medicine, 238(4), 433–441. 10.1177/1535370213477600 PMC4457439 23760009

[eph70452-bib-0004] Boeno, F. P. , Ramis, T. R. , Munhoz, S. V. , Farinha, J. B. , Moritz, C. E. J. , Leal‐Menezes, R. , Ribeiro, J. L. , Christou, D. D. , & Reischak‐Oliveira, A. (2020). Effect of aerobic and resistance exercise training on inflammation, endothelial function and ambulatory blood pressure in middle‐aged hypertensive patients. Journal of Hypertension, 38(12), 2501–2509. 10.1097/HJH.0000000000002581 32694343

[eph70452-bib-0005] Broxterman, R. M. , Witman, M. A. , Trinity, J. D. , Groot, H. J. , Rossman, M. J. , Park, S. Y. , Malenfant, S. , Gifford, J. R. , Kwon, O. S. , Park, S. H. , Jarrett, C. L. , Shields, K. L. , Hydren, J. R. , Bisconti, A. V. , Owan, T. , Abraham, A. , Tandar, A. , Lui, C. Y. , Smith, B. R. , & Richardson, R. S. (2019). Strong relationship between vascular function in the coronary and brachial arteries: A clinical coming of age for the updated flow‐mediated dilation test?. Hypertension, 74(1), 208–215. 10.1161/HYPERTENSIONAHA.119.12881 PMC6716528 31055952

[eph70452-bib-0006] Bull, F. C. , Al‐Ansari, S. S. , Biddle, S. , Borodulin, K. , Buman, M. P. , Cardon, G. , Carty, C. , Chaput, J.‐P. , Chastin, S. , Chou, R. , Dempsey, P. C. , DiPietro, L. , Ekelund, U. , Firth, J. , Friedenreich, C. M. , Garcia, L. , Gichu, M. , Jago, R. , Katzmarzyk, P. T. , … Willumsen, J. F. (2020). World Health Organization 2020 guidelines on physical activity and sedentary behaviour. British Journal of Sports Medicine, 54(24), 1451–1462. 10.1136/bjsports-2020-102955 PMC7719906 33239350

[eph70452-bib-0007] Cattermole, G. N. , Leung, P. M. , Ho, G. Y. , Lau, P. W. , Chan, C. P. , Chan, S. S. , Smith, B. E. , Graham, C. A. , & Rainer, T. H. (2017). The normal ranges of cardiovascular parameters measured using the ultrasonic cardiac output monitor. Physiological Reports, 5(6), e13195. 10.14814/phy2.13195 PMC5371563 28320891

[eph70452-bib-0008] Cortez‐Cooper, M. Y. , Anton, M. M. , DeVan, A. E. , Neidre, D. B. , Cook, J. N. , & Tanaka, H. (2008). The effects of strength training on central arterial compliance in middle‐aged and older adults. European Journal of Preventive Cardiology, 15(2), 149–155. 10.1097/HJR.0b013e3282f02fe2 18391640

[eph70452-bib-0009] Dawkins, T. G. , Curry, B. A. , Drane, A. L. , Lord, R. N. , Richards, C. , Brown, M. , Pugh, C. J. A. , Lodge, F. , Yousef, Z. , Stembridge, M. , & Shave, R. E. (2020). Stimulus‐specific functional remodeling of the left ventricle in endurance and resistance‐trained men. American Journal of Physiology‐Heart and Circulatory Physiology, 319(3), H632–H641. 10.1152/ajpheart.00233.2020 32772543

[eph70452-bib-0010] Dawkins, T. G. , Curry, B. A. , Drane, A. L. , Lord, R. N. , Richards, C. T. , Lodge, F. M. , Yousef, Z. , Pugh, C. J. A. , Shave, R. E. , & Stembridge, M. (2022). Evidence of region‐specific right ventricular functional adaptation in endurance‐trained men in response to an acute volume infusion. Experimental Physiology, 107(1), 6–15. 10.1113/EP089882 34743381

[eph70452-bib-0011] de Oliveira, G. V. , Mendes Cordeiro, E. , Volino‐Souza, M. , Rezende, C. , Conte‐Junior, C. A. , & Silveira Alvares, T. (2020). Flow‐mediated dilation in healthy young individuals is impaired after a single resistance exercise session. International Journal of Environmental Research and Public Health, 17(14), 5194. 10.3390/ijerph17145194 PMC7400374 32708408

[eph70452-bib-0012] Figueroa, A. , Park, S. Y. , Seo, D. Y. , Sanchez‐Gonzalez, M. A. , & Baek, Y. H. (2011). Combined resistance and endurance exercise training improves arterial stiffness, blood pressure, and muscle strength in postmenopausal women. Menopause, 18(9), 980–984. 10.1097/gme.0b013e3182135442 21540753

[eph70452-bib-0013] Flück, D. , Ainslie, P. N. , Bain, A. R. , Wildfong, K. W. , Morris, L. E. , & Fisher, J. P. (2017). Extra‐and intracranial blood flow regulation during the cold pressor test: Influence of age. Journal of Applied Physiology, 123(5), 1071–1080. 10.1152/japplphysiol.00224.2017 PMC5792099 28663374

[eph70452-bib-0014] Green, D. J. , Hopman, M. T. , Padilla, J. , Laughlin, M. H. , & Thijssen, D. H. (2017). Vascular adaptation to exercise in humans: Role of hemodynamic stimuli. Physiological Reviews, 97(2), 495–528. 10.1152/physrev.00014.2016 PMC5539408 28151424

[eph70452-bib-0015] Green, D. J. , Jones, H. , Thijssen, D. , Cable, N. T. , & Atkinson, G. (2011). Flow‐mediated dilation and cardiovascular event prediction: Does nitric oxide matter?. Hypertension, 57(3), 363–369. 10.1161/HYPERTENSIONAHA.110.167015 21263128

[eph70452-bib-0016] Hornby‐Foster, I. , Richards, C. T. , Drane, A. L. , Lodge, F. M. , Stembridge, M. , Lord, R. N. , Davey, H. , Yousef, Z. , & Pugh, C. J. A. (2025). Resistance‐and endurance‐trained young men display comparable carotid artery strain parameters that are superior to untrained men. European Journal of Applied Physiology, 125(1), 131–144. 10.1007/s00421-024-05598-w PMC11753328 39361034

[eph70452-bib-0017] Kawano, H. , Tanimoto, M. , Yamamoto, K. , Sanada, K. , Gando, Y. , Tabata, I. , Higuchi, M. , & Miyachi, M. (2008). Resistance‐training in men is associated with increased arterial stiffness and blood pressure but does not adversely affect endothelial function as measured by arterial reactivity to the cold pressor test. Experimental Physiology, 93(2), 296–302. 10.1113/expphysiol.2007.039867 17911355

[eph70452-bib-0018] Lacombe, J. , Armstrong, M. E. , Wright, F. L. , & Foster, C. (2019). The impact of physical activity and an additional behavioural risk factor on cardiovascular disease, cancer and all‐cause mortality: A systematic review. BMC Public Health, 19(1), 900. 10.1186/s12889-019-7030-8 PMC6615183 31286911

[eph70452-bib-0019] Lambiase, M. J. , Dorn, J. , Thurston, R. C. , & Roemmich, J. N. (2014). Flow‐mediated dilation and exercise blood pressure in healthy adolescents. Journal of Science and Medicine in Sport, 17(4), 425–429. 10.1016/j.jsams.2013.05.015 PMC4020997 23809838

[eph70452-bib-0020] Leloup, A. J. , Van Hove, C. E. , Heykers, A. , Schrijvers, D. M. , De Meyer, G. R. , & Fransen, P. (2015). Elastic and muscular arteries differ in structure, basal NO production and voltage‐gated Ca^2+^‐channels. Frontiers in Physiology, 6, 375. 10.3389/fphys.2015.00375 PMC4678217 26696904

[eph70452-bib-0021] Londono, F. J. , Klopmeijer, T. S. , Georgakopoulos, D. , Lovett, E. G. , Van Bortel, L. , & Segers, P. (2014). Carotid haemodynamics during sympathetic nervous system stimulation via handgrip and cold pressor testing in young healthy subjects: A feasibility study. Artery Research, 8(4), 178.

[eph70452-bib-0022] Matthews, K. A. , Owens, J. F. , Kuller, L. H. , Sutton‐Tyrrell, K. , Lassila, H. C. , & Wolfson, S. K. (1998). Stress‐induced pulse pressure change predicts women's carotid atherosclerosis. Stroke; A Journal of Cerebral Circulation, 29(8), 1525–1530. 10.1161/01.str.29.8.1525 9707187

[eph70452-bib-0023] Mitchell, G. F. , van Buchem, M. A. , Sigurdsson, S. , Gotal, J. D. , Jonsdottir, M. K. , Kjartansson, Ó. , Garcia, M. , Aspelund, T. , Harris, T. B. , Gudnason, V. , & Launer, L. J. (2011). Arterial stiffness, pressure and flow pulsatility and brain structure and function: The age, gene/environment susceptibility–reykjavik study. Brain, 134(11), 3398–3407. 10.1093/brain/awr253 PMC3212721 22075523

[eph70452-bib-0024] Miura, H. , Takahashi, Y. , Maki, Y. , & Sugino, M. (2015). Effects of exercise training on arterial stiffness in older hypertensive females. European Journal of Applied Physiology, 115(9), 1847–1854. 10.1007/s00421-015-3168-y 25869875

[eph70452-bib-0025] Miyachi, M. (2013). Effects of resistance‐training on arterial stiffness: A meta‐analysis. British Journal of Sports Medicine, 47(6), 393–396. 10.1136/bjsports-2012-090488 22267567

[eph70452-bib-0026] Mortensen, S. P. , Nyberg, M. , Gliemann, L. , Thaning, P. , Saltin, B. , & Hellsten, Y. (2014). Exercise training modulates functional sympatholysis and α‐adrenergic vasoconstrictor responsiveness in hypertensive and normotensive individuals. The Journal of Physiology, 592(14), 3063–3073. 10.1113/jphysiol.2014.273722 PMC4214660 24860173

[eph70452-bib-0027] Otsuki, T. , Maeda, S. , Iemitsu, M. , Saito, Y. , Tanimura, Y. , Ajisaka, R. , & Miyauchi, T. (2007). Vascular endothelium‐derived factors and arterial stiffness in strength‐and endurance‐trained men. American Journal of Physiology‐Heart and Circulatory Physiology, 292(2), H786–H791. 10.1152/ajpheart.00678.2006 16997889

[eph70452-bib-0028] Pugh, C. J. A. , Stone, K. J. , Stöhr, E. J. , McDonnell, B. J. , Thompson, J. E. S. , Talbot, J. S. , Wakeham, D. J. , Cockcroft, J. R. , & Shave, R. (2018). Carotid artery wall mechanics in young males with high cardiorespiratory fitness. Experimental Physiology, 103(9), 1277–1286. 10.1113/EP087067 29959801

[eph70452-bib-0029] Rubenfire, M. , Rajagopalan, S. , & Mosca, L. (2000). Carotid artery vasoreactivity in response to sympathetic stress correlates with coronary disease risk and is independent of wall thickness. Journal of the American College of Cardiology, 36(7), 2192–2197. 10.1016/s0735-1097(00)01021-4 11127460

[eph70452-bib-0030] Seals, D. R. , Jablonski, K. L. , & Donato, A. J. (2011). Aging and vascular endothelial function in humans. Clinical Science, 120(9), 357–375. 10.1042/CS20100476 PMC3482987 21244363

[eph70452-bib-0031] Seals, D. R. , Nagy, E. E. , & Moreau, K. L. (2019). Aerobic exercise training and vascular function with ageing in healthy men and women. The Journal of Physiology, 597(19), 4901–4914. 10.1113/JP277764 PMC6773490 31077372

[eph70452-bib-0032] Stone, R. M. , Ainslie, P. N. , Kerstens, T. P. , Wildfong, K. W. , & Tymko, M. M. (2019). Sex differences in the circulatory responses to an isocapnic cold pressor test. Experimental Physiology, 104(3), 295–305. 10.1113/EP087232 30578582

[eph70452-bib-0033] Sun, H. , Zhang, Y. , & Shi, L. (2024). Advances in exercise‐induced vascular adaptation: Mechanisms, models, and methods. Frontiers in Bioengineering and Biotechnology, 12, 1370234. 10.3389/fbioe.2024.1370234 PMC10917942 38456010

[eph70452-bib-0034] Talbot, J. S. , Lord, R. N. , Wakeham, D. J. , Dawkins, T. G. , Curry, B. A. , Brown, M. , Lodge, F. M. , & Pugh, C. J. A. (2020). The influence of habitual endurance exercise on carotid artery strain and strain rate in young and middle‐aged men. Experimental Physiology, 105(8), 1396–1407. 10.1113/EP088384 32578897

[eph70452-bib-0035] Tarumi, T. , & Zhang, R. (2018). Cerebral blood flow in normal aging adults: Cardiovascular determinants, clinical implications, and aerobic fitness. Journal of Neurochemistry, 144(5), 595–608. 10.1111/jnc.14234 PMC5874160 28986925

[eph70452-bib-0036] Thijssen, D. H. , de Groot, P. , Kooijman, M. , Smits, P. , & Hopman, M. T. (2006). Sympathetic nervous system contributes to the age‐related impairment of flow‐mediated dilation of the superficial femoral artery. American Journal of Physiology‐Heart and Circulatory Physiology, 291(6), H3122–H3129. 10.1152/ajpheart.00240.2006 16844924

[eph70452-bib-0037] Thijssen, D. H. J. , Black, M. A. , Pyke, K. E. , Padilla, J. , Atkinson, G. , Harris, R. A. , Parker, B. , Widlansky, M. E. , Tschakovsky, M. E. , & Green, D. J. (2011). Assessment of flow‐mediated dilation in humans: A methodological and physiological guideline. American Journal of Physiology‐Heart and Circulatory Physiology, 300(1), H2–H12. 10.1152/ajpheart.00471.2010 PMC3023245 20952670

[eph70452-bib-0038] Tousoulis, D. , Davies, G. , Tentolouris, C. , Crake, T. , & Toutouzas, P. (1997). Inhibition of nitric oxide synthesis during the cold pressor test in patients with coronary artery disease. American Journal of Cardiology, 79(12), 1676–1679. 10.1016/s0002-9149(97)00222-1 9202363

[eph70452-bib-0039] Tymko, M. M. , Kerstens, T. P. , Wildfong, K. W. , & Ainslie, P. N. (2017). Cerebrovascular response to the cold pressor test–the critical role of carbon dioxide. Experimental Physiology, 102(12), 1647–1660. 10.1113/EP086585 28925529

[eph70452-bib-0040] Vallbracht‐Israng, K. B. , Morguet, A. , & Schwimmbeck, P. L. (2007). Correlation of epicardial and systemic flow‐mediated vasodilation in patients with atypical angina but no evidence of atherosclerotic disease. Canadian Journal of Cardiology, 23(13), 1054–1060. 10.1016/s0828-282x(07)70873-5 PMC2651929 17985007

[eph70452-bib-0041] Van Bortel, L. M. , Duprez, D. , Starmans‐Kool, M. J. , Safar, M. E. , Giannattasio, C. , Cockcroft, J. , & Thuillez, C. (2002). Clinical applications of arterial stiffness, Task Force III: Recommendations for user procedures. American journal of hypertension, 15(5), 445–452. 10.1016/s0895-7061(01)02326-3 12022247

[eph70452-bib-0042] Van Mil, A. C. , Hartman, Y. , Van Oorschot, F. , Heemels, A. , Bax, N. , Dawson, E. A. , Hopkins, N. , Hopman, M. T. , Green, D. J. , Oxborough, D. L. , & Thijssen, D. H. (2017). Correlation of carotid artery reactivity with cardiovascular risk factors and coronary artery vasodilator responses in asymptomatic, healthy volunteers. Journal of Hypertension, 35(5), 1026–1034. 10.1097/HJH.0000000000001274 28129249

[eph70452-bib-0043] Yang, W. I. , Kang, K. W. , Lee, H. Y. , Shim, C. Y. , Hong, G. R. , Chung, N. , & Ha, J. W. (2018). Incremental predictive value of carotid arterial strain in patients with stroke. The International Journal of Cardiovascular Imaging, 34(6), 893–902. 10.1007/s10554-018-1301-9 29327164

[eph70452-bib-0044] Yu, C. C.‐W. , McManus, A. M. , So, H.‐K. , Chook, P. , Au, C.‐T. , Li, A. M. , Kam, J. T. C. , So, R. C. H. , Lam, C. W.‐K. , Chan, I. H.‐S. , & Sung, R. Y. T. (2016). Effects of resistance‐training on cardiovascular health in non‐obese active adolescents. World Journal of Clinical Pediatrics, 5(3), 293. 10.5409/wjcp.v5.i3.293 PMC4978622 27610345

